# Early Prediction of Mortality Risk in Acute Respiratory Distress Syndrome: Systematic Review and Meta-Analysis

**DOI:** 10.2196/70537

**Published:** 2025-05-20

**Authors:** Ruimin Tan, Chen Ge, Zhe Li, Yating Yan, He Guo, Wenjing Song, Qiong Zhu, Quansheng Du

**Affiliations:** 1 School of Clinical Medical North China University of Science and Technology Tangshan China; 2 Critical Care Department Hebei General Hospital Shijiazhuang China; 3 School of Graduate Chengde Medical University Chengde China; 4 Critical Care Department Handan Central Hospital Handan China; 5 Critical Care Department Hebei Medical University Shijiazhuang China; 6 Department of Orthopaedics The People’s Hospital of Shizhu Chongqing China

**Keywords:** acute respiratory distress syndrome, mortality, machine learning, systematic review, meta-analysis

## Abstract

**Background:**

Acute respiratory distress syndrome (ARDS) is a life-threatening condition associated with high mortality rates. Despite advancements in critical care, reliable early prediction methods for ARDS-related mortality remain elusive. Accurate risk assessment is crucial for timely intervention and improved patient outcomes. Machine learning (ML) techniques have emerged as promising tools for mortality prediction in patients with ARDS, leveraging complex clinical datasets to identify key prognostic factors. However, the efficacy of ML-based models remains uncertain. This systematic review aims to assess the value of ML models in the early prediction of ARDS mortality risk and to provide evidence supporting the development of simplified, clinically applicable ML-based scoring tools for prognosis.

**Objective:**

This study systematically reviewed available literature on ML-based ARDS mortality prediction models, primarily aiming to evaluate the predictive performance of these models and compare their efficacy with conventional scoring systems. It also sought to identify limitations and provide insights for improving future ML-based prediction tools.

**Methods:**

A comprehensive literature search was conducted across PubMed, Web of Science, the Cochrane Library, and Embase, covering publications from inception to April 27, 2024. Studies developing or validating ML-based ARDS mortality predicting models were considered for inclusion. The methodological quality and risk of bias were assessed using the Prediction Model Risk of Bias Assessment Tool (PROBAST). Subgroup analyses were performed to explore heterogeneity in model performance based on dataset characteristics and validation approaches.

**Results:**

In total, 21 studies involving a total of 31,291 patients with ARDS were included. The meta-analysis demonstrated that ML models achieved relatively high predictive performance. In the training datasets, the pooled concordance index (C-index) was 0.84 (95% CI 0.81-0.86), while for in-hospital mortality prediction, the pooled C-index was 0.83 (95% CI 0.81-0.86). In the external validation datasets, the pooled C-index was 0.81 (95% CI 0.78-0.84), and the corresponding value for in-hospital mortality prediction was 0.80 (95% CI 0.77-0.84). ML models outperformed traditional scoring tools, which demonstrated lower predictive performance. The pooled area under the receiver operating characteristic curve (ROC-AUC) for standard scoring systems was 0.7 (95% CI 0.67-0.72). Specifically, 2 widely used clinical scoring systems, the Sequential Organ Failure Assessment (SOFA) and Simplified Acute Physiology Score II (SAPS-II), demonstrated ROC-AUCs of 0.64 (95% CI 0.62-0.67) and 0.70 (95% CI 0.66-0.74), respectively.

**Conclusions:**

ML-based models exhibited superior predictive accuracy over conventional scoring tools, suggesting their potential use in early ARDS mortality risk assessment. However, further research is needed to refine these models, improve their interpretability, and enhance their clinical applicability. Future efforts should focus on developing simplified, efficient, and user-friendly ML-based prediction tools that integrate seamlessly into clinical workflows. Such advancements may facilitate the early identification of high-risk patients, enabling timely interventions and personalized, risk-based prevention strategies to improve ARDS outcomes.

## Introduction

Acute respiratory distress syndrome (ARDS) is a serious and complex condition characterized by acute, diffuse inflammatory lung injury leading to rapid progress to acute respiratory failure, posing significant challenges in patient management [1]. This syndrome can be triggered by various pulmonary and extrapulmonary pathogenic factors, including severe infections, shock, trauma, and extensive burns, all of which contribute to its onset and severity [2]. ARDS has a profound impact with high morbidity and mortality rates, positioning it as a key concern in critical care medicine. Findings from the largest international cohort study on ARDS, LUNG SAFE, revealed that approximately 10.4% of patients admitted to intensive care units were diagnosed with this condition. Alarmingly, the mortality rate among these patients can reach as high as 45% [3,4]. This staggering mortality rate is intricately linked to the complex pathophysiological mechanisms underlying ARDS, which involve not only lung injuries but also multisystem injuries, inflammatory responses, dysregulation of coagulation pathways, and other interacting biological processes [5]. Current pharmacological treatments for ARDS focus on alleviating pulmonary edema and inflammation, promoting vasodilation, and enhancing the repair processes of epithelial, endothelial, and extracellular matrix tissues [6]. These interventions are crucial for reducing mortality rates and improving the overall prognosis for affected patients. Consequently, the early identification of potential mortality risks in patients with ARDS, coupled with timely and effective interventions, holds the promise of reversing adverse clinical outcomes and improving survival rates [7].

Currently, no reliable scoring methods are available for predicting mortality in patients with ARDS within the realm of clinical practice [8]. While scoring tools such as the Sequential Organ Failure Assessment (SOFA) and Acute Physiology and Chronic Health Evaluation II (APACHE II) show certain predictive value regarding mortality in critically ill patients, their actual effectiveness in real-world clinical settings remains a topic of controversy among health care professionals [9]. This debate significantly limits the practical application of these scoring systems in everyday medical scenarios.

In recent years, machine learning (ML) technology has gained considerable traction and is now extensively used in the prediction, diagnosis, and treatment of critical and severe diseases [10]. A comprehensive meta-analysis conducted by Islam et al [11] has demonstrated that ML methods exhibit superior predictive performance compared with existing scoring systems, particularly in the context of sepsis onset. Similarly, Tacconelli et al [12] made significant strides by developing the BLOOMY score through ML techniques, which is effective in predicting both 14-day and 6-month mortality rates for people with bloodstream infections.

In light of these advancements, researchers have turned their attention to the development of ML-based predictive models specifically aimed at forecasting mortality in patients with ARDS [13]. However, despite these efforts, systematic evidence robustly supporting the predictive performance of these models is still lacking, poising a considerable challenge to the creation of simplified clinical tools that could be widely adopted in practice [14]. Therefore, this study was conducted with the primary objective of evaluating the effectiveness of ML-based predictive models for ARDS mortality, ultimately providing valuable evidence to support the development and refinement of simplified and efficient clinical tools that could enhance patient care and outcomes.

## Methods

### Protocol and Registration

The study was carried out following the 2020 PRISMA (Preferred Reporting Items for Systematic Reviews and Meta-Analyses) statement (Multimedia Appendix 1) [15] and was preregistered with PROSPERO (International Prospective Register of Systematic Reviews, ID: CRD42024549357) [16].

### Study Eligibility

The inclusion criteria for this study included cohort, case-control, and cross-sectional studies. The study population consisted of individuals diagnosed with ARDS according to internationally recognized guidelines at the time of the study with no restrictions on the underlying causes of ARDS. As this study aimed to systematically review the effectiveness of ML-based tools in predicting ARDS mortality, the observation group included patients with ARDS who died during the follow-up period, regardless of the specific causes of death. The primary outcome measure was the concordance index (C-index), which reflects the overall accuracy of the predictive models. In addition, the frequency of variables used in model development was considered a secondary outcome measure.

Studies were excluded if they were expert opinions, meta-analyses, guidelines, reviews, or unpublished conference abstracts. Research that did not involve the construction of a predictive model and instead examined risk factors was also excluded. Furthermore, studies lacking outcome measures necessary for assessing model accuracy, such as accuracy, C-statistic, C-index, receiver operating characteristic (ROC) curve, sensitivity (SEN), specificity (SPE), *F*_1_-score, recall, precision, confusion matrix, calibration curve, or diagnostic 2×2 tables, were not considered. In addition, studies with an insufficient sample size, defined as fewer than 20 participants, were excluded from this review. Finally, papers that were not published in English were excluded.

### Literature Search Strategy

Up until April 27, 2024, we performed a thorough search across PubMed, Web of Science, the Cochrane Library, and Embase. Both MeSH (Medical Subject Headings) and free-text terms were used for searching with no regard to publication year or region. To identify and acquire pertinent material, the references of the included research were also retrospectively searched. Details are provided in Table S1 in Multimedia Appendix 2.

### Literature Screening and Data Extraction

After being imported into EndNote (version 20; Clarivate), retrieved studies were screened to remove duplicates. Following this, the titles and abstracts of the retrieved publications were evaluated to identify preliminarily eligible original studies. Then, full texts of these studies were downloaded to screen qualified studies. For data extraction, we formulated a standardized electronic data extraction form to capture essential information, including title, DOI, first author, publication year, country of the authors, study type, patient source, disease background, and follow-up duration. The data extraction and literature screening procedures were completed independently by 2 researchers. Cross-checking was completed to ensure consistency. In case of discrepancies, a third researcher was consulted to discuss and determine whether the study should be included. The Cohen κ coefficient was used to assess the consistency of the 2 researchers’ assessments. The Cohen κ coefficient for the final screening results was 0.823, indicating a high degree of agreement between their assessments.

### Risk of Bias Assessment for Included Studies

To assess the risk of bias in the included original studies, the Prediction Model Risk of Bias Assessment Tool (PROBAST) was used. PROBAST encompasses four domains with multiple questions: participants, predictors, outcomes, and statistical analysis, which collectively reflect the overall risk of bias and overall applicability. Each of these domains contains specific questions, with 3 possible responses for each question: “Yes” or “Probably yes” (low risk of bias), “No” or “Probably no” (high risk of bias), and “No information.” If all domains were judged as low risk, the total risk of bias was assessed as low, if at least 1 domain was judged as high risk, the total risk was rated as high. Using PROBAST, 2 researchers independently appraised the risk of bias. After completion, they cross-checked the assessments, and a third researcher was involved to resolve discrepancies and reach a final consensus in cases of disagreement.

### Statistical Analysis

C-index, an indicator of the overall accuracy of ML models, was meta-analyzed. For original studies lacking a 95% CI or SE for the C-index, the SE was estimated based on the methods described by Debray et al [17]. For the meta-analysis of the C-index, a random-effects model was prioritized due to the varied parameters and variables in ML models. Furthermore, a bivariate mixed-effects model was applied to analyze SEN and SPE. SEN and SPE were then meta-analyzed based on diagnostic 2×2 tables. However, as most original studies did not report diagnostic 2×2 tables, we calculated these metrics using SEN, SPE, and precision based on the number of cases. All related meta-analyses were done in R (version 4.2.0, R Development Core Team).

### Ethical Considerations

Our study was a systematic review that used nonidentifiable, secondary data from published studies. According to institutional policies, no ethics review was required.

## Results

### Results of Literature Screening

**Figure 1:**
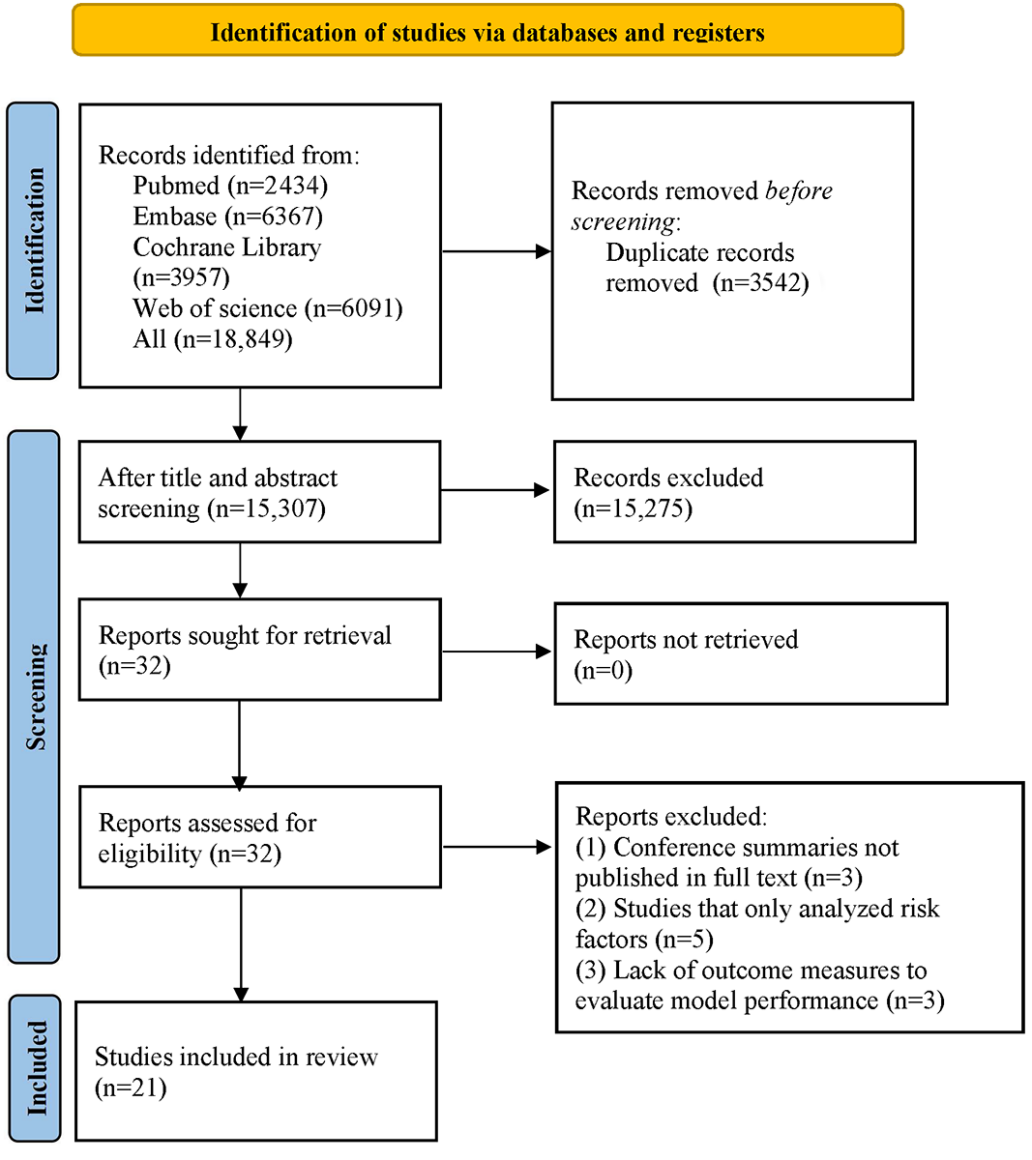
PRISMA (Preferred Reporting Items for Systematic Reviews and Meta-Analyses) 2020 flow diagram.

Overall, the search yielded 18,849 papers, including 2434 from PubMed, 6091 from Web of Science, 3957 from Cochrane Library, and 6367 from Embase. After removing 3542 duplicates and excluding studies irrelevant to the topic per title and abstract, 32 papers were identified for further review. These preliminarily eligible studies were downloaded and thoroughly read, resulting in 21 original studies [18-38] involving a population of 31,291 patients with ARDS being included (PRISMA flow diagram is presented in Figure 1).

### Basic Characteristics of Included Studies

Among the 21 studies included a total of 31,291 patients with ARDS were analyzed. All 21 studies were cohort studies with 6 [18,20,24,32,33,37] being prospective cohort studies and 15 [19,21-23,25-31,34-36,38] being retrospective cohort studies. Of these, 7 [19,24,28,31,32,34,37] studies were conducted in single-center settings, 8 [18,20-22,27,33,36,38] were multicenter studies, and 7 [23,25-27,29,30,35] used data from registry databases, primarily the Medical Information Mart for Intensive Care III (MIMIC-III) database. The majority of the multicenter research was carried out in developed areas such as Europe and North America. A total of 11 studies [18,19,21,22,27,30,31,33,34,36,38] provided detailed information on the disease background, 4 studies [19,21,36,38] focused on patients with ARDS receiving extracorporeal membrane oxygenation therapy, and 3 studies [22,30,33] focused on those undergoing mechanical ventilation. In terms of mortality outcomes, 18 studies [18-23,25,27-35,37,38] focused on in-hospital mortality, while 6 studies [19,23-26,36] examined out-of-hospital mortality. Out of the 21 studies, 12 [18,21,22,25,26,28-30,33-35,38] included independent validation sets, 6 [21,22,25,29,30,38] of these used external validation sets, while 10 [18,21,26,28-30,33-35,38] used internally validated datasets based on random sampling. The studies evaluated 5 different models with a primary focus on logistic regression. Other models included multilayer perceptron, random forest, Extreme Gradient Boosting (XGBoost), and radial basis function. In total, 9 studies [18,19,24,25,27,30,32,33,38] compared ML models with conventional scoring tools, 6 studies [18,19,25,27,30,38] provided SOFA scores, 4 [25,27,30,38] provided SAPS II scores, 1 [19] provided APACHE II scores, and 1 [30] provided APACHE IV scores (Table S2 in Multimedia Appendix 3).

### Risk of Bias Assessment

**Figure 2:**
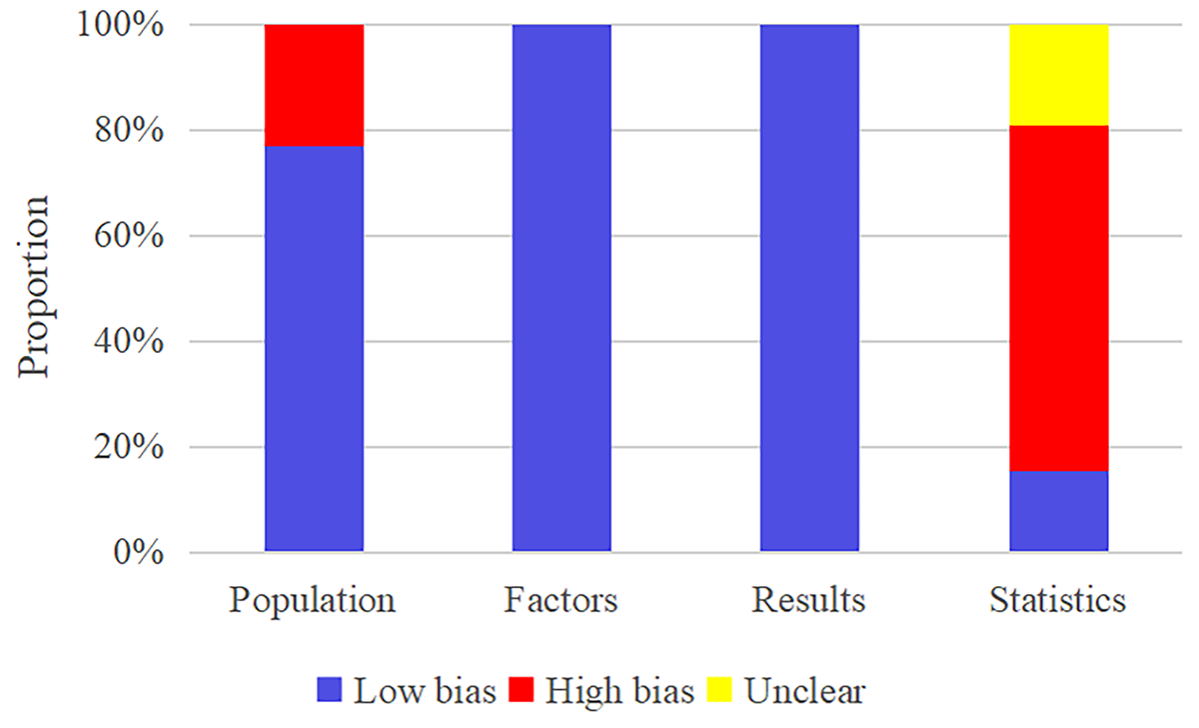
Risk of bias assessment of the included studies.

We assessed the risk of bias for the 26 ML models included in our review. The results indicated that 6 models were based on retrospective cohort studies, resulting in a high risk of bias concerning study participants. As all studies were cohort studies, there was no high risk of bias regarding predictors. Since predicted mortality was the outcome, no complex assessment was needed for outcome evaluation, suggesting a low risk of bias in this domain. In terms of statistical analysis, 16 models did not meet the criterion that the number of death cases in the training set should be 20 times the number of modeling variables, nor did they meet the criterion that the number of cases in the validation set (Events Per Variable >20) should exceed 100. These models were therefore rated as having a high risk of bias. In addition, 6 models had inappropriate methods for handling missing values, indicating a high risk of bias. Similarly, 6 models also used unsuitable methods for model fitting. The risk of bias assessment results is detailed in Figure 2. In addition, to assess potential publication bias, we conducted Egger tests for various scores, training sets, and validation sets, respectively. The results of the publication bias and Egger tests are presented in Figures S1-S6 in Multimedia Appendix 4.

### Meta-Analysis

**Figure 3:**
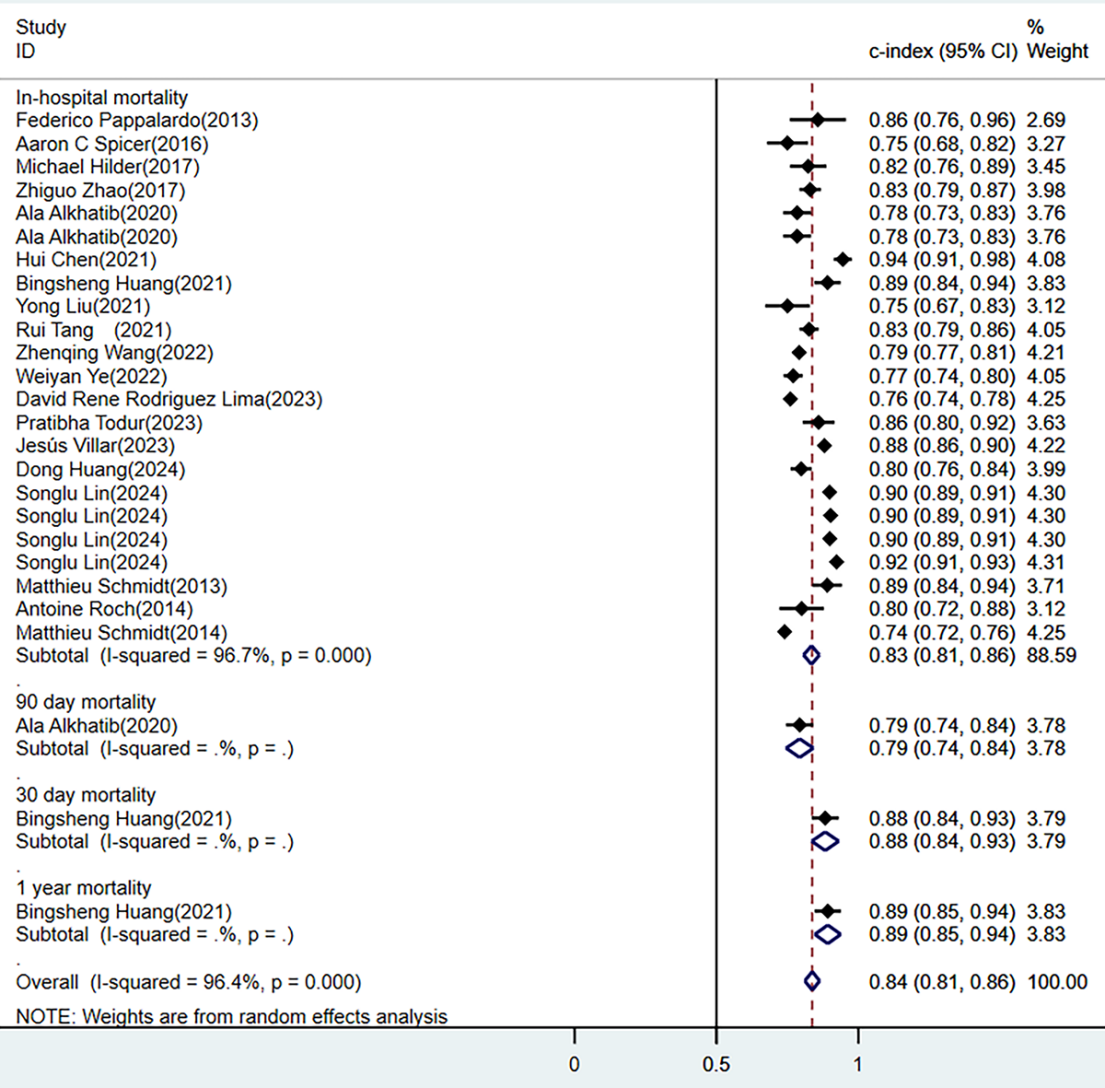
Forest plot of meta-analysis for C-index in the training set [18,20-27,29-38].

**Figure 4:**
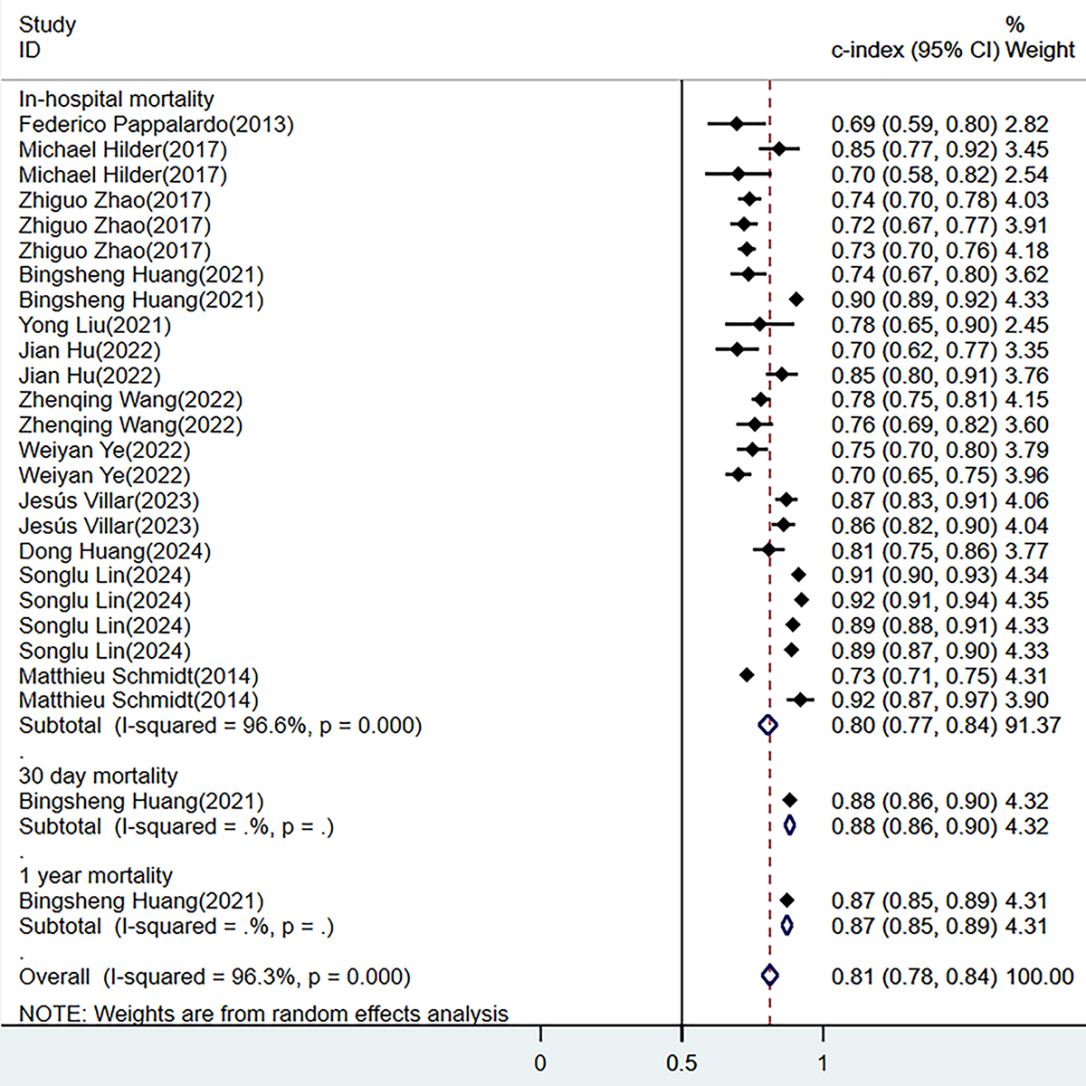
Forest plot of meta-analysis for C-index in the validation set [18,21,22,25,26,28-30,33-36].

**Figure 5:**
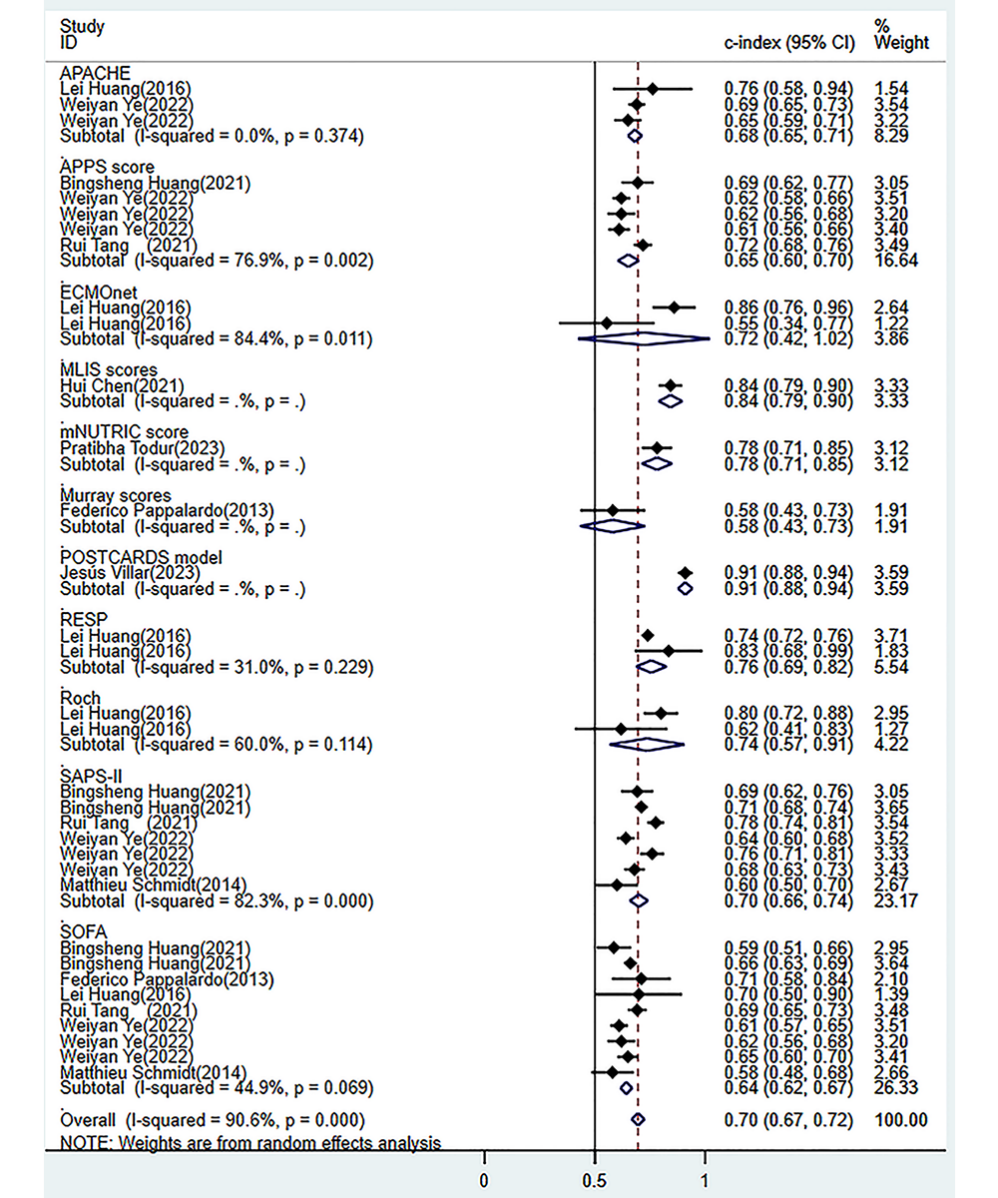
Forest plot of meta-analysis for C-index of each scoring tool in the validation set [18,19,24,25,27,30,32,33,38].

A total of 26 models were constructed in the training sets, yielding a pooled C-index of 0.84 (95% CI 0.81-0.86; Figure 3 [18,20-27,29-38]). Forest plot summarizing the predictive performance of various models for in-hospital, 30-day, 90-day, and 1-year mortality using the C-index (95% CI). The overall pooled estimate indicates strong predictive ability with substantial heterogeneity across studies [18,20-38]. Among these, 23 models predicted in-hospital mortality, yielding a pooled area under the ROC curve (ROC-AUC) of 0.83 (95% CI 0.81-0.86). In the validation sets, 26 models were also constructed, yielding a pooled C-index of 0.81 (95% CI 0.78-0.84; Figure 4 [18,21,22,25,26,28-30,33-36]). Forest plot displaying the C-index (95% CI) of different predictive models for in-hospital, 30-day, and 1-year mortality. The pooled analysis suggests moderate to high predictive performance with heterogeneity present among the included studies [18,21,22,25,26,28-30,33-36]. Among these, 24 models predicted in-hospital mortality, yielding a pooled ROC-AUC of 0.80 (95% CI 0.77-0.84). A total of 13 cohorts validated the SOFA score, resulting in a pooled ROC-AUC of 0.64 (95% CI 0.62-0.67) and 11 cohorts validated the Simplified Acute Physiology Score II (SAPS-II) score, showing a pooled ROC-AUC of 0.70 (95% CI 0.66-0.74; Figure 5 [18,19,24,25,27,30,32,33,38]). Forest plot comparing the C-index (95% CI) of various severity scoring systems, including APACHE, SAPS-II, SOFA, and other models, for mortality prediction. The overall predictive performance varies across different scoring systems, with notable heterogeneity in some subgroups [18,19,24,25,27,30,32,33,36].

### Predictors

In the newly constructed ML models, we extracted all predictors. Key predictors included age, lactate level, oxygenation index, creatinine level, platelet count, albumin, white blood cell count, body temperature, and immune function impairment (details are provided in Table S3 in Multimedia Appendix 5).

### SEN Analysis

To assess the robustness of the findings, a SEN analysis was conducted. We assessed whether individual studies significantly altered the overall pooled C-index by sequentially excluding each study. The results remained consistent, suggesting that no single study disproportionately affected the conclusions. We stratified studies based on key factors such as model type, dataset size, and outcome definitions to determine whether heterogeneity was driven by specific subgroups. The findings indicated that some model categories exhibited lower heterogeneity than others. The results of the SEN analysis are uploaded as Figures S7-S9 in Multimedia Appendix 4.

## Discussion

### Principal Findings

This meta-analysis found that while the SOFA and SAPS-II scores exhibited certain predictive performance for ARDS mortality, their overall effectiveness remains a concern. We acknowledge that traditional scoring systems offer well-established, interpretable tools for clinical decision-making with a long history of validation in various critical care settings. However, they may have limitations in capturing complex, nonlinear relationships within heterogeneous patient populations. For instance, SOFA and SAPS-II scores rely on predefined variables and thresholds, which may not adequately adapt to dynamic patient conditions or account for interactions between multiple risk factors. These limitations can lead to reduced accuracy in certain subgroups, such as patients with atypical presentations or those with multimorbidity.

Conversely, ML models excel in identifying complex patterns in high-dimensional data and can provide more individualized risk assessments. They may offer superior predictive performance in cases where traditional models fail to fully capture patient heterogeneity, such as sepsis, which involves rapidly evolving pathophysiological changes. In addition, ML models can complement traditional scoring systems by integrating additional predictive features and allowing real-time updates based on new clinical data. On this basis, the new ML models analyzed in this study demonstrated superior predictive performance with a pooled C-index of 0.81 (95% CI 0.78-0.84) in the validation sets and that of 0.80 (95% CI 0.77-0.84) for in-hospital mortality.

### Comparison With Previous Work

Previous studies have explored early prediction methods for ARDS mortality risk, but they mainly focused on identifying risk factors. For example, in a study by Jayasimhan et al [39], dead space ventilation was identified as an independent risk factor for ARDS mortality. Similarly, Terpstra et al [40] confirmed in their review that a reduced protein C level is associated with increased ARDS incidence and mortality. Other research has shown that an elevated level of angiopoietin-2 can independently predict mortality risk in patients with ARDS [41]. However, in clinical practice, mortality risk assessment based solely on individual risk factors remains vague and poses significant challenges.

Researchers have also explored other predictive methods in hopes of achieving more accurate mortality predictions for ARDS. In a meta-analysis of cardiac biomarkers in ARDS, it was confirmed that several cardiac biomarkers, including N-terminal pro-B-type natriuretic peptide and B-type natriuretic peptide, were associated with higher mortalities [42]. Zeng et al [43] found that angiopoietin-2 had certain prognostic value for patients with ARDS, with a ROC-AUC of 0.83, SEN of 0.69, and SPE of 0.81. However, the study exhibited considerable heterogeneity in prognostic analysis, and angiopoietin-2 was only a single predictor. Some researchers have attempted to predict short-term mortality in patients with ARDS using computed tomography. Unfortunately, the results showed SEN of 0.62 (95% CI 0.30-0.88) and SPE of 0.76 (95% CI 0.57-0.89) [44], indicating significant limitations in using computed tomography for short-term mortality prediction in patients with ARDS. Building on this foundation, our study summarized ML models constructed from commonly used and interpretable risk factors and biomarkers, which demonstrated superior predictive performance for ARDS mortality of these models. Therefore, future research could focus on developing simple and widely applicable scoring tools using ML methods and these multivariable clinical factors.

Rashid et al [45] performed a systematic review of the application of ML in ARDS. However, their study included only 2 original studies on mortality prediction, and these studies did not reach conclusions regarding the accuracy of mortality prediction. Tran et al [46] included 12 studies that explored the management, prediction, and classification of ARDS based on ML methods. However, they did not address the applicability or provide definitive conclusions regarding these approaches. In our study, we presented a more comprehensive evidence summary and concluded that new ML models have favorable predictive performance for ARDS mortality, with a pooled C-index of up to 0.80 for in-hospital mortality.

### Limitations

This study provides evidence-based support for ML-based models for the prediction of mortality risk in ARDS. However, limitations that should be considered are as follows. First, even though we systematically searched several databases, only a limited number of studies met the inclusion criteria. Second, in constructing ML models, diverse modeling methods were used; however, due to limited evidence, this study analyzed only a small subset of studies on certain ML methods, which restricted the depth of our discussion. Third, external validation is crucial for assessing model performance across different populations, reducing the risk of overfitting, and ensuring broader clinical applicability. However, challenges such as data availability, patient heterogeneity, and institutional differences often hinder external validation efforts in this field. The studies included in this meta-analysis primarily relied on internal validation using random samples, which may limit the interpretability of the results. Future studies should incorporate multicenter datasets with diverse demographic and clinical characteristics, and prospective validation using external datasets from different institutions is necessary to assess real-world performance. In addition, to improve clinical adoption, future models should incorporate interpretable techniques, such as Shapley additive Explanation values or attention mechanisms, to improve transparency. Lastly, the causes of ARDS are highly varied and come from a variety of illness backgrounds. However, the limited evidence included in this study prevents a more in-depth exploration of the different etiological backgrounds of ARDS. Regarding the representativeness of the included studies, we acknowledge that differences in study design, patient populations, and geographic distribution may affect the generalizability of the findings. To improve reproducibility, we recommend that future studies adopt standardized reporting guidelines, such as TRIPOD+AI (Transparent Reporting of a Multivariable Prediction Model for Individual Prognosis or Diagnosis+Artificial Intelligence), to ensure comprehensive and transparent documentation of data preprocessing, model development, and validation procedures. In addition, promoting data and code sharing could facilitate independent verification of findings.

### Conclusions

ML-based models for predicting ARDS mortality risk demonstrated relatively strong predictive performance, surpassing that of commonly used individual scoring tools. However, our findings are based on limited evidence, which imposes certain limitations on its value for guiding clinical practice. Therefore, clinicians should exercise caution in interpreting and applying these findings. Future studies are expected to further develop or update efficient, simplified, and clinically applicable ML-based ARDS mortality risk prediction tools. Such tools could facilitate early identification of ARDS mortality risk and aid in formulating individualized, personalized, risk-based prevention strategies.

## Data Availability

The datasets generated or analyzed during this study are available from the corresponding author upon reasonable request.

## Appendix

Multimedia Appendix 1 PRISMA (Preferred Reporting Items for Systematic Reviews and Meta-Analyses) 2020 checklist.

Multimedia Appendix 2 Search strategy and result.

Multimedia Appendix 3 Characteristics of included studies.

Multimedia Appendix 4 Supplementary Figures.

Multimedia Appendix 5 Predictive factors.

## Abbreviations

APACHE II: Acute Physiology and Chronic Health Evaluation II
ARDS: acute respiratory distress syndrome
MeSH: Medical Subject Headings
MIMIC-III: Medical Information Mart for Intensive Care III
ML: machine learning
PRISMA: Preferred Reporting Items for Systematic Reviews and Meta-Analyses
PROBAST: Prediction Model Risk of Bias Assessment Tool
PROSPERO: International Prospective Register of Systematic Reviews
ROC: receiver operating characteristic
ROC-AUC: area under the receiver operating characteristic curve
SAPS-II: Simplified Acute Physiology Score II
SEN: sensitivity
SOFA: Sequential Organ Failure Assessment
SPE: specificity
TRIPOD+AI: Transparent Reporting of a Multivariable Prediction Model for Individual Prognosis or Diagnosis+Artificial Intelligence
XGBoost: Extreme Gradient Boosting
