# Supplementary material for: Early Prediction of Mortality Risk in Acute Respiratory Distress Syndrome: Systematic Review and Meta-Analysis
Source: J Med Internet Res. 2025 May 20;27:e70537. doi: 10.2196/70537 (PMC12134695; doi:10.2196/70537)
Supplement: Multimedia Appendix 2 [file jmir_v27i1e70537_app2.docx]

**S1 Table Search strategy and result**

**1.Pubmed**

| Search number | Query | Results |
| --- | --- | --- |
| #1 | Respiratory Distress Syndrome [Mesh] | 41,352 |
| #2 | ((((((((((((Respiratory Distress Syndrome[Title/Abstract]) OR (Respiratory Distress Syndrome[Title/Abstract])) OR (Shock Lung[Title/Abstract])) OR (Acute Respiratory Distress Syndrome[Title/Abstract])) OR (Pediatric Respiratory Distress Syndrome[Title/Abstract])) OR (Adult Respiratory Distress Syndrome[Title/Abstract])) OR (Acute-respiratory distress syndrome[Title/Abstract])) OR (breathing distress syndrome[Title/Abstract])) OR (lung distress syndrome[Title/Abstract])) OR (pulmonary distress syndrome[Title/Abstract])) OR (RDS[Title/Abstract])) OR (respiration distress syndrome[Title/Abstract])) OR (ARDS[Title/Abstract]) | 49,228 |
| #3 | (Respiratory Distress Syndrome [Mesh]) OR (((((((((((((Respiratory Distress Syndrome[Title/Abstract]) OR (Respiratory Distress Syndrome[Title/Abstract])) OR (Shock Lung[Title/Abstract])) OR (Acute Respiratory Distress Syndrome[Title/Abstract])) OR (Pediatric Respiratory Distress Syndrome[Title/Abstract])) OR (Adult Respiratory Distress Syndrome[Title/Abstract])) OR (Acute-respiratory distress syndrome[Title/Abstract])) OR (breathing distress syndrome[Title/Abstract])) OR (lung distress syndrome[Title/Abstract])) OR (pulmonary distress syndrome[Title/Abstract])) OR (RDS[Title/Abstract])) OR (respiration distress syndrome[Title/Abstract])) OR (ARDS[Title/Abstract])) | 67,700 |
| #4 | machine learning[MeSH Terms] | 67,057 |
| #5 | ((((((((((((((((((((((((((((((machine learning[Title/Abstract]) OR (Transfer Learning[Title/Abstract])) OR (Deep learning[Title/Abstract])) OR (Ensemble Learning[Title/Abstract])) OR (artificial intelligence[Title/Abstract])) OR (random forest[Title/Abstract])) OR (neural network[Title/Abstract])) OR (neural networks[Title/Abstract])) OR (K-Nearest Neighbor[Title/Abstract])) OR (CNN[Title/Abstract])) OR (Support vector machine[Title/Abstract])) OR (SVM[Title/Abstract])) OR (Gradient Boosting Machine[Title/Abstract])) OR (Nomogram[Title/Abstract])) OR (XGBoost[Title/Abstract])) OR (Adaboost[Title/Abstract])) OR (Decision tree[Title/Abstract])) OR (ResNet-50[Title/Abstract])) OR (ResNet[Title/Abstract])) OR (AlexNet[Title/Abstract])) OR (VGGNet[Title/Abstract])) OR (GoogLeNet[Title/Abstract])) OR (Naive Bayesian[Title/Abstract])) OR (Multilayer perceptron[Title/Abstract])) OR (Bayesian network[Title/Abstract])) OR (Radiomics[Title/Abstract])) OR (Radiomic[Title/Abstract])) OR (Prediction model[Title/Abstract])) OR (Risk model[Title/Abstract])) OR (risk factors[Title/Abstract])) OR (predictors[Title/Abstract]) | 1,219,473 |
| #6 | (machine learning[MeSH Terms]) OR (((((((((((((((((((((((((((((((machine learning[Title/Abstract]) OR (Transfer Learning[Title/Abstract])) OR (Deep learning[Title/Abstract])) OR (Ensemble Learning[Title/Abstract])) OR (artificial intelligence[Title/Abstract])) OR (random forest[Title/Abstract])) OR (neural network[Title/Abstract])) OR (neural networks[Title/Abstract])) OR (K-Nearest Neighbor[Title/Abstract])) OR (CNN[Title/Abstract])) OR (Support vector machine[Title/Abstract])) OR (SVM[Title/Abstract])) OR (Gradient Boosting Machine[Title/Abstract])) OR (Nomogram[Title/Abstract])) OR (XGBoost[Title/Abstract])) OR (Adaboost[Title/Abstract])) OR (Decision tree[Title/Abstract])) OR (ResNet-50[Title/Abstract])) OR (ResNet[Title/Abstract])) OR (AlexNet[Title/Abstract])) OR (VGGNet[Title/Abstract])) OR (GoogLeNet[Title/Abstract])) OR (Naive Bayesian[Title/Abstract])) OR (Multilayer perceptron[Title/Abstract])) OR (Bayesian network[Title/Abstract])) OR (Radiomics[Title/Abstract])) OR (Radiomic[Title/Abstract])) OR (Prediction model[Title/Abstract])) OR (Risk model[Title/Abstract])) OR (risk factors[Title/Abstract])) OR (predictors[Title/Abstract])) | 1,224,570 |
| #7 | Mortality[MeSH Terms] | 426,665 |
| #8 | (((((((Mortality[Title/Abstract]) OR (Mortalities[Title/Abstract])) OR (Case Fatality Rate[Title/Abstract])) OR (Case Fatality Rates[Title/Abstract])) OR (Death Rate[Title/Abstract])) OR (Death Rates[Title/Abstract])) OR (Survival[Title/Abstract])) OR (Death[Title/Abstract]) | 2,734,852 |
| #9 | (Mortality[MeSH Terms]) OR ((((((((Mortality[Title/Abstract]) OR (Mortalities[Title/Abstract])) OR (Case Fatality Rate[Title/Abstract])) OR (Case Fatality Rates[Title/Abstract])) OR (Death Rate[Title/Abstract])) OR (Death Rates[Title/Abstract])) OR (Survival[Title/Abstract])) OR (Death[Title/Abstract])) | 2,864,140 |
| #10 | (((Respiratory Distress Syndrome [Mesh]) OR (((((((((((((Respiratory Distress Syndrome[Title/Abstract]) OR (Respiratory Distress Syndrome[Title/Abstract])) OR (Shock Lung[Title/Abstract])) OR (Acute Respiratory Distress Syndrome[Title/Abstract])) OR (Pediatric Respiratory Distress Syndrome[Title/Abstract])) OR (Adult Respiratory Distress Syndrome[Title/Abstract])) OR (Acute-respiratory distress syndrome[Title/Abstract])) OR (breathing distress syndrome[Title/Abstract])) OR (lung distress syndrome[Title/Abstract])) OR (pulmonary distress syndrome[Title/Abstract])) OR (RDS[Title/Abstract])) OR (respiration distress syndrome[Title/Abstract])) OR (ARDS[Title/Abstract]))) AND ((machine learning[MeSH Terms]) OR (((((((((((((((((((((((((((((((machine learning[Title/Abstract]) OR (Transfer Learning[Title/Abstract])) OR (Deep learning[Title/Abstract])) OR (Ensemble Learning[Title/Abstract])) OR (artificial intelligence[Title/Abstract])) OR (random forest[Title/Abstract])) OR (neural network[Title/Abstract])) OR (neural networks[Title/Abstract])) OR (K-Nearest Neighbor[Title/Abstract])) OR (CNN[Title/Abstract])) OR (Support vector machine[Title/Abstract])) OR (SVM[Title/Abstract])) OR (Gradient Boosting Machine[Title/Abstract])) OR (Nomogram[Title/Abstract])) OR (XGBoost[Title/Abstract])) OR (Adaboost[Title/Abstract])) OR (Decision tree[Title/Abstract])) OR (ResNet-50[Title/Abstract])) OR (ResNet[Title/Abstract])) OR (AlexNet[Title/Abstract])) OR (VGGNet[Title/Abstract])) OR (GoogLeNet[Title/Abstract])) OR (Naive Bayesian[Title/Abstract])) OR (Multilayer perceptron[Title/Abstract])) OR (Bayesian network[Title/Abstract])) OR (Radiomics[Title/Abstract])) OR (Radiomic[Title/Abstract])) OR (Prediction model[Title/Abstract])) OR (Risk model[Title/Abstract])) OR (risk factors[Title/Abstract])) OR (predictors[Title/Abstract])))) AND ((Mortality[MeSH Terms]) OR ((((((((Mortality[Title/Abstract]) OR (Mortalities[Title/Abstract])) OR (Case Fatality Rate[Title/Abstract])) OR (Case Fatality Rates[Title/Abstract])) OR (Death Rate[Title/Abstract])) OR (Death Rates[Title/Abstract])) OR (Survival[Title/Abstract])) OR (Death[Title/Abstract]))) | 2,434 |

**2.Cochrane**

| Search number | Query | Results |
| --- | --- | --- |
| #1 | MeSH descriptor: [Respiratory Distress Syndrome] explode all trees | 3590 |
| #2 | (Respiratory Distress Syndrome):ti,ab,kw OR (Respiratory Distress Syndromes):ti,ab,kw OR (Shock Lung):ti,ab,kw OR (Acute Respiratory Distress Syndrome):ti,ab,kw OR (Pediatric Respiratory Distress Syndrome):ti,ab,kw | 8094 |
| #3 | (Adult Respiratory Distress Syndrome):ti,ab,kw OR (Acute-respiratory distress syndrome):ti,ab,kw OR (breathing distress syndrome):ti,ab,kw OR (lung distress syndrome):ti,ab,kw OR (pulmonary distress syndrome):ti,ab,kw | 6228 |
| #4 | (RDS):ti,ab,kw OR (respiration distress syndrome):ti,ab,kw OR (ARDS):ti,ab,kw | 4859 |
| #5 | #1 or #2 or #3 or #4 | 9370 |
| #6 | MeSH descriptor: [Respiratory Distress Syndrome] explode all trees | 3590 |
| #7 | (machine learning):ti,ab,kw OR (Transfer Learning):ti,ab,kw OR (Deep learning):ti,ab,kw OR (Ensemble Learning):ti,ab,kw OR (artificial intelligence):ti,ab,kw | 7210 |
| #8 | (random forest):ti,ab,kw OR (neural network):ti,ab,kw OR (neural networks):ti,ab,kw OR (K-Nearest Neighbor):ti,ab,kw OR (CNN):ti,ab,kw | 4497 |
| #9 | (Support vector machine):ti,ab,kw OR (SVM):ti,ab,kw OR (Gradient Boosting Machine):ti,ab,kw OR (Nomogram):ti,ab,kw OR (XGBoost):ti,ab,kw | 2467 |
| #10 | (Adaboost):ti,ab,kw OR (Decision tree):ti,ab,kw OR (ResNet-50):ti,ab,kw OR (ResNet):ti,ab,kw OR (AlexNet):ti,ab,kw | 1053 |
| #11 | (VGGNet):ti,ab,kw OR (GoogLeNet):ti,ab,kw OR (Naive Bayesian):ti,ab,kw OR (Multilayer perceptron):ti,ab,kw OR (Bayesian network):ti,ab,kw | 518 |
| #12 | (Radiomics):ti,ab,kw OR (Radiomic):ti,ab,kw OR (Prediction model):ti,ab,kw OR (Risk model):ti,ab,kw OR (risk factors):ti,ab,kw | 121224 |
| #13 | (predictors):ti,ab,kw | 24425 |
| #14 | #6 or #7 or #8 or #9 or #10 or #11 or #12 or #13 | 151392 |
| #15 | MeSH descriptor: [Respiratory Distress Syndrome] explode all trees | 3590 |
| #16 | (Mortality):ti,ab,kw OR (Mortalities):ti,ab,kw OR (Case Fatality Rate):ti,ab,kw OR (Case Fatality Rates):ti,ab,kw OR (Death Rate):ti,ab,kw | 138079 |
| #17 | (Death Rates):ti,ab,kw OR (Survival):ti,ab,kw OR (Death):ti,ab,kw | 192528 |
| #18 | #15 or #16 or #17 | 256816 |
| #19 | #5 and #14 and #18 | 3957 |

**3.Embase**

| Search number | Query | Results |
| --- | --- | --- |
| #1 | 'respiratory distress syndrome'/exp | 109190 |
| #2 | 'respiratory distress syndrome'/exp OR 'respiratory distress syndrome' OR (('respiratory'/exp OR respiratory) AND ('distress'/exp OR distress) AND ('syndrome'/exp OR syndrome)) OR 'respiratory distress syndromes':ab,ti OR 'shock lung':ab,ti OR 'acute respiratory distress syndrome':ab,ti OR 'pediatric respiratory distress syndrome':ab,ti OR 'adult respiratory distress syndrome':ab,ti OR 'acute-respiratory distress syndrome':ab,ti OR 'breathing distress syndrome':ab,ti OR 'lung distress syndrome':ab,ti OR 'pulmonary distress syndrome':ab,ti OR rds:ab,ti OR 'respiration distress syndrome':ab,ti OR ards:ab,ti | 141818 |
| #3 | #1 OR #2 | 141818 |
| #4 | 'machine learning'/exp | 470838 |
| #5 | machine learning OR 'transfer learning':ab,ti OR 'deep learning':ab,ti OR 'ensemble learning':ab,ti OR 'artificial intelligence':ab,ti OR 'random forest':ab,ti OR 'neural network':ab,ti OR 'neural networks':ab,ti OR 'k-nearest neighbor':ab,ti OR cnn:ab,ti OR 'support vector machine':ab,ti OR svm:ab,ti OR 'gradient boosting machine':ab,ti OR nomogram:ab,ti OR xgboost:ab,ti OR adaboost:ab,ti OR 'decision tree':ab,ti OR 'resnet 50':ab,ti OR resnet:ab,ti OR alexnet:ab,ti OR vggnet:ab,ti OR googlenet:ab,ti OR 'naive bayesian':ab,ti OR 'multilayer perceptron':ab,ti OR 'bayesian network':ab,ti OR radiomics:ab,ti OR radiomic:ab,ti OR 'prediction model':ab,ti OR 'risk model':ab,ti OR 'risk factors':ab,ti OR predictors:ab,ti | 2130500 |
| #6 | #4 OR #5 | 2322264 |
| #7 | 'mortality'/exp | 1459330 |
| #8 | 'mortality'/exp OR mortality OR mortalities:ab,ti OR 'case fatality rate':ab,ti OR 'case fatality rates':ab,ti OR 'death rate':ab,ti OR 'death rates':ab,ti OR survival:ab,ti OR death:ab,ti | 4303491 |
| #9 | #7 OR #8 | 4303491 |
| #10 | #3 AND #6 AND #9 | 6367 |

**4.Web of science**

| Search number | Query | Results |
| --- | --- | --- |
| #1 | Respiratory Distress Syndrome (Topic) OR Respiratory Distress Syndromes (Topic) OR Shock Lung (Topic) OR Acute Respiratory Distress Syndrome (Topic) OR Pediatric Respiratory Distress Syndrome (Topic) OR Adult Respiratory Distress Syndrome (Topic) OR Acute-respiratory distress syndrome (Topic) OR breathing distress syndrome (Topic) OR lung distress syndrome (Topic) OR pulmonary distress syndrome (Topic) OR RDS (Topic) OR respiration distress syndrome (Topic) OR ARDS (Topic) | 80838 |
| #2 | machine learning (Topic) OR Transfer Learning (Topic) OR Deep learning (Topic) OR Ensemble Learning (Topic) OR artificial intelligence (Topic) OR random forest (Topic) OR neural network (Topic) OR neural networks (Topic) OR K-Nearest Neighbor (Topic) OR CNN (Topic) OR Support vector machine (Topic) OR SVM (Topic) OR Gradient Boosting Machine (Topic) OR Nomogram (Topic) OR XGBoost (Topic) OR Adaboost (Topic) OR Decision tree (Topic) OR ResNet-50 (Topic) OR ResNet (Topic) OR AlexNet (Topic) OR VGGNet (Topic) OR GoogLeNet (Topic) OR Naive Bayesian (Topic) OR Multilayer perceptron (Topic) OR Bayesian network (Topic) OR Radiomics (Topic) OR Radiomic (Topic) OR Prediction model (Topic) OR Risk model (Topic) OR risk factors (Topic) OR predictors (Topic) | 5012937 |
| #3 | Mortality (Topic) OR Mortalities (Topic) OR Case Fatality Rate (Topic) OR Case Fatality Rates (Topic) OR Death Rate (Topic) OR Death Rates (Topic) OR Survival (Topic) OR Death (Topic) | 3545729 |
| #4 | #1 AND #2 AND #3 | 6091 |
