# Supplementary material for: Early Prediction of Mortality Risk in Acute Respiratory Distress Syndrome: Systematic Review and Meta-Analysis
Source: J Med Internet Res. 2025 May 20;27:e70537. doi: 10.2196/70537 (PMC12134695; doi:10.2196/70537)

**Supplementary Figure**

**Figure S1** Funnel plot of publication bias for each type of score.


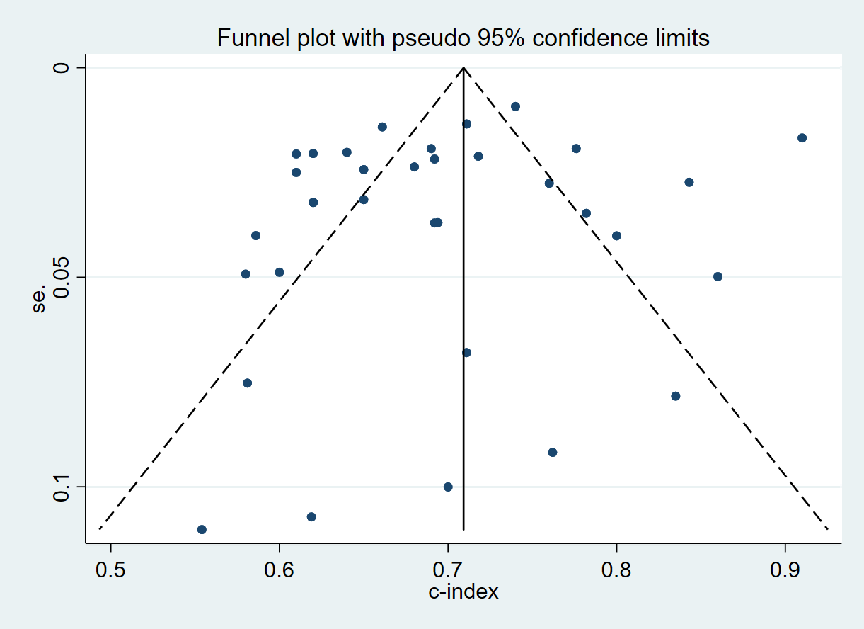


**Figure S2** Egger's test results for each rating category.


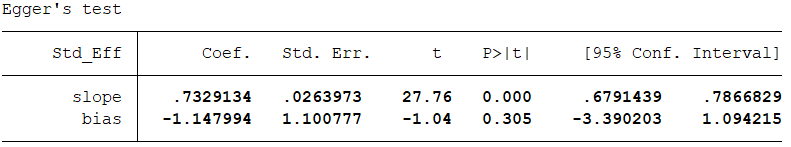


**Figure S3** Funnel plot of publication bias in the training set.


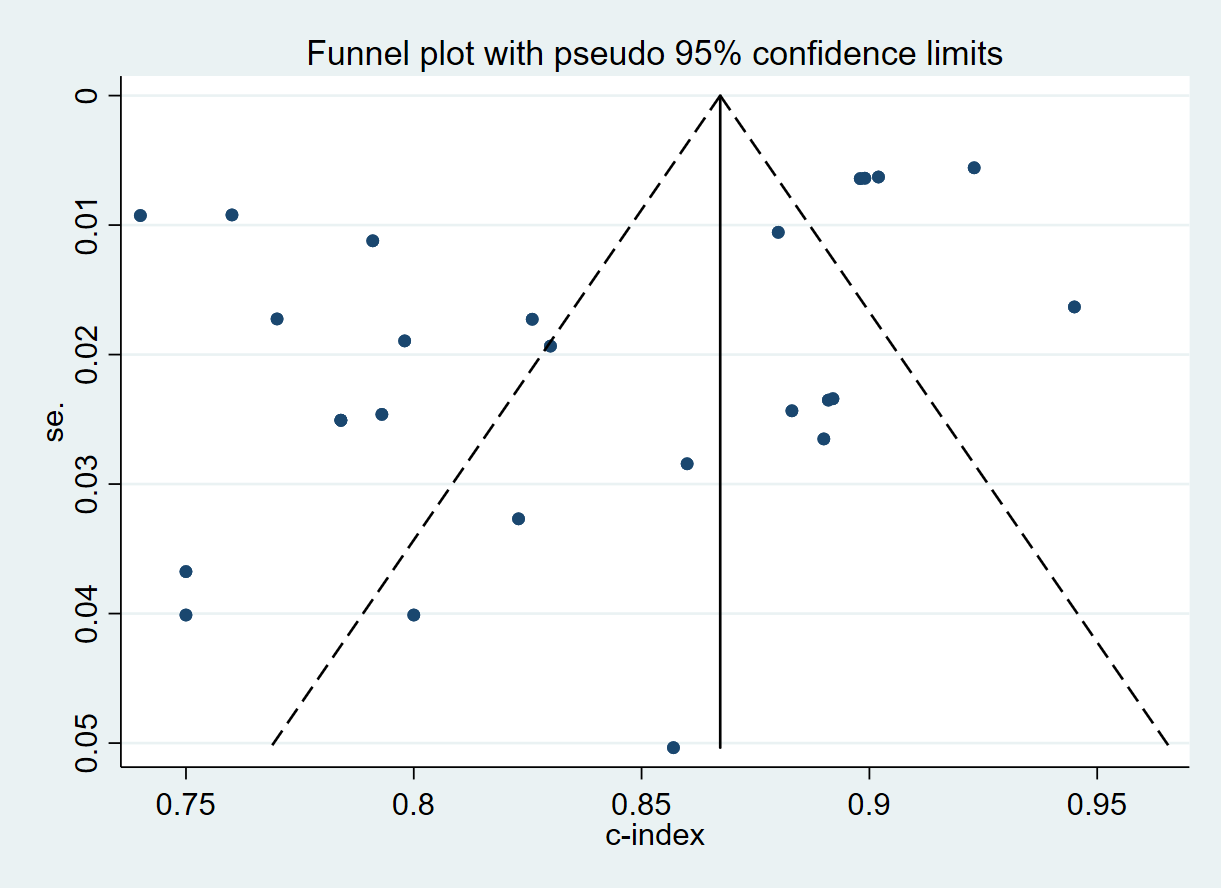


**Figure S4** Egger's test results in the training set.


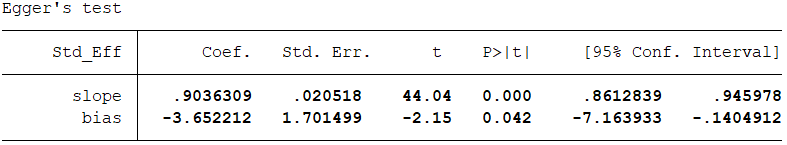


**Figure S5** Funnel plot of publication bias in the validation set.


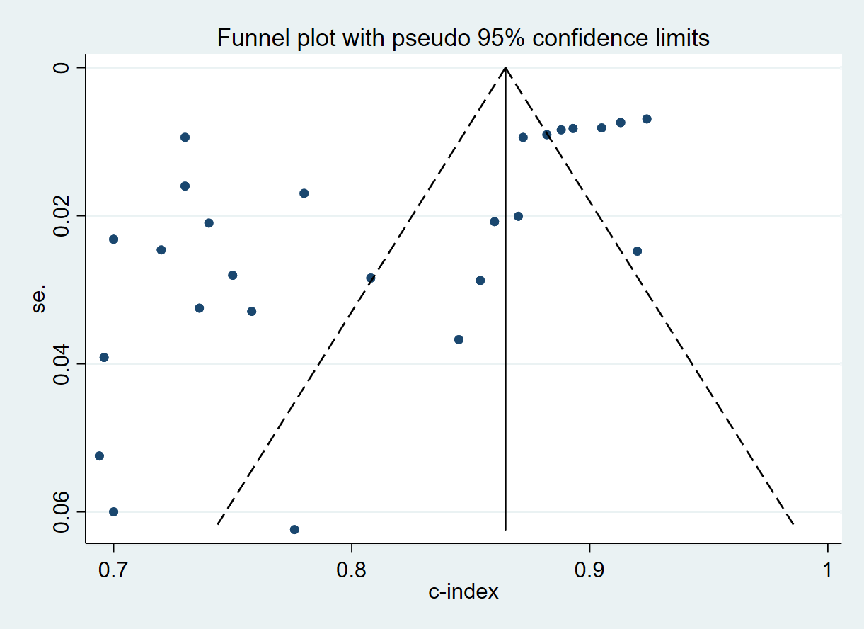


**Figure S6** Egger's test results in the validation set.


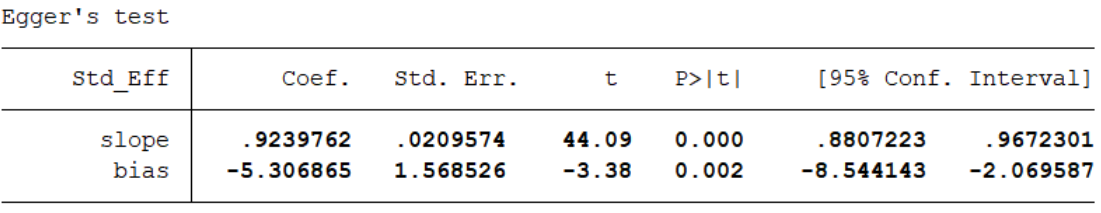


**Figure S7** Results of the sensitivity analysis of the various scores.


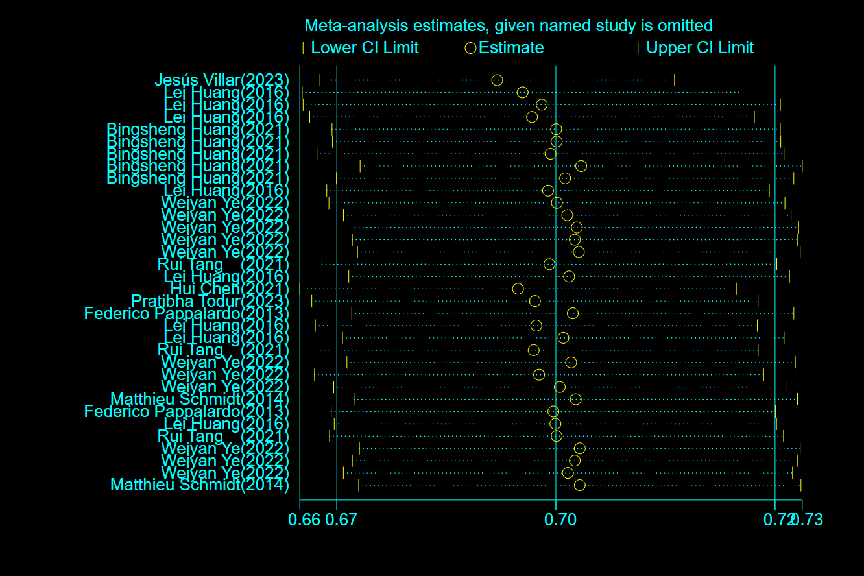


**Figure S8** Results of sensitivity analysis in the training set.


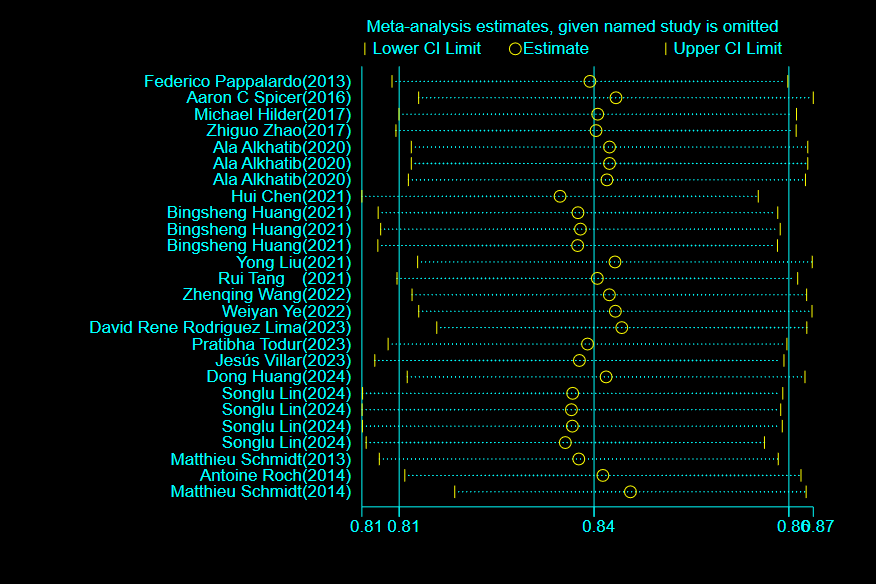


**Figure S9** Results of sensitivity analysis in the validation set.


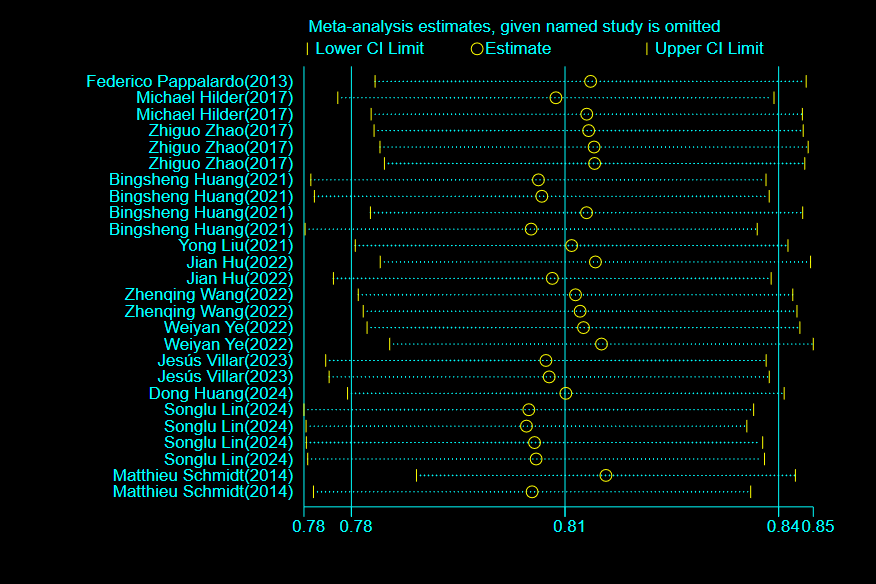

Supplement: Multimedia Appendix 4 [file jmir_v27i1e70537_app4.doc]
