# Supplementary material for: Early Prediction of Mortality Risk in Acute Respiratory Distress Syndrome: Systematic Review and Meta-Analysis
Source: J Med Internet Res. 2025 May 20;27:e70537. doi: 10.2196/70537 (PMC12134695; doi:10.2196/70537)
Supplement: Multimedia Appendix 5 [file jmir_v27i1e70537_app5.docx]

**S3 Table** **Predictive factors**

| No | factors | Number | No | factors | Number |
| --- | --- | --- | --- | --- | --- |
| 1 | Bilirubin value | 2 | 40 | Heart failure | 3 |
| 2 | Systemic mean arterial pressure | 5 | 41 | Renal failure | 3 |
| 3 | Hematocrit value | 3 | 42 | SAPS II | 3 |
| 4 | PreECMO hospital length of stay | 5 | 43 | Immune function impairment | 10 |
| 5 | Creatinine level | 12 | 44 | TBIL | 3 |
| 6 | OI | 1 | 45 | Respiratory rate | 11 |
| 7 | Cancer/HSCT history | 1 | 46 | INR | 3 |
| 8 | PHa | 10 | 47 | AaDO2 | 3 |
| 9 | Lactate concentration | 14 | 48 | Height | 1 |
| 10 | Platelet concentration | 11 | 49 | Sodium | 1 |
| 11 | Age | 34 | 50 | Potassium | 1 |
| 12 | APACHE III score | 4 | 51 | Severity of ARDS | 1 |
| 13 | Plasma IL8 | 4 | 52 | mNUTRIC score | 1 |
| 14 | SPD | 6 | 53 | DP | 1 |
| 15 | Sex | 3 | 54 | Pplat | 5 |
| 16 | Comorbidity score | 3 | 55 | Chronic cardiovascular diseases | 2 |
| 17 | SOFA | 5 | 56 | Chronic respiratory diseases | 2 |
| 18 | PaO2/FiO2 | 21 | 57 | Lymphocyte | 2 |
| 19 | RDW | 8 | 58 | D-dimer | 2 |
| 20 | NLR | 1 | 59 | Procalcitonin | 2 |
| 21 | PAR | 1 | 60 | Days of MV | 4 |
| 22 | MLIS | 1 | 61 | Prone positioning | 1 |
| 23 | WBC | 10 | 62 | PEEP | 9 |
| 24 | Albumin | 11 | 63 | Influenza pneumonia | 1 |
| 25 | FiO2 | 7 | 64 | Diagnosis | 3 |
| 26 | PaO2 | 7 | 65 | Central nervous system | 3 |
| 27 | Heart rate | 7 | 66 | Dysfunction | 3 |
| 28 | Temperature | 18 | 67 | Acute nonpulmonary infection | 3 |
| 29 | BMI | 9 | 68 | Neuromuscular blockade agents | 3 |
| 30 | LDH | 4 | 69 | Nitric oxide use | 3 |
| 31 | HRCT | 2 | 70 | Bicarbonate infusion | 3 |
| 32 | Etiology | 2 | 71 | Cardiac arrest | 3 |
| 33 | BUN | 4 | 72 | PaCO2 | 3 |
| 34 | Acute kidney failure | 1 | 73 | Peak inspiratory pressure | 3 |
| 35 | Sepsis | 1 | 74 | APS III score | 8 |
| 36 | Admission type | 1 | 75 | SBP | 8 |
| 37 | Organ failure | 4 | 76 | MBP | 8 |
| 38 | SaO2% | 1 | 77 | DBP | 8 |
| 39 | Hemoglobin | 4 | 78 | GCS score | 8 |
